# Thermosonication for the Production of Sulforaphane Rich Broccoli Ingredients

**DOI:** 10.3390/biom11020321

**Published:** 2021-02-20

**Authors:** Sajad Shokri, Hema Jegasothy, Mary Ann Augustin, Netsanet Shiferaw Terefe

**Affiliations:** 1 CSIRO Agriculture and Food, 671 Sneydes Road, Werribee, VIC 3030, Australia; s.shokri@shirazu.ac.ir (S.S.); Hemalatha.jegasothy@csiro.au (H.J.); maryann.augustin@csiro.au (M.A.A.); 2 Department of Food Hygiene and Control, Shiraz University, Shiraz 71454, Iran

**Keywords:** broccoli, thermosonication, mild heat, sulforaphane, myrosinase, glucoraphanin

## Abstract

A large proportion of broccoli biomass is lost during primary production, distribution, processing, and consumption. This biomass is rich in polyphenols and glucosinolates and can be used for the production of bioactive rich ingredients for food and nutraceutical applications. This study evaluated thermosonication (TS) (18 kHz, 0.6 W/g, 40–60 °C, 3–7 min) for the pre-treatment of broccoli florets to enhance enzymatic conversion of glucoraphanin into the bioactive sulforaphane. TS significantly increased sulforaphane yield, despite a decrease in myrosinase activity with increasing treatment intensity. The highest sulforaphane yield of ~2.9 times that of untreated broccoli was observed for broccoli thermosonicated for 7 min at 60 °C, which was 15.8% higher than the corresponding yield for thermal processing without sonication (TP) at the same condition. This was accompanied by increase in the residual level of glucoraphanin (~1.8 and 2.3 time respectively after TP and TS at 60 °C for 7 min compared to control samples) indicating that treatment-induced release of bound glucoraphanin from the cell wall matrix and improved accessibility could be at least partially responsible for the enhanced sulforaphane yield. The result indicates the potential of TS for the conversion of broccoli biomass into high sulforaphane broccoli-based ingredients.

## 1. Introduction

A large amount of vegetable biomass is wasted during the primary production, distribution, processing, and consumption of vegetables. This includes the potentially edible parts of the plant left on the field in normal harvest practice, wastage in the fresh market due to postharvest quality deterioration, and trimmings produced during consumption and processing. A significant proportion of the produce is also deemed second grade i.e., not suitable for the premium market, especially in the developed world mainly due to cosmetic market specifications. Broccoli is among the vegetables with the highest wastage in Australia. Only 40% of the broccoli plant material is harvested as the leaves and the stem are usually plowed back into the field or used as animal feed. Of the edible part, less than 50% is sold as a premium produce with 12 to 30% considered second grade and 28% wasted in the fresh market. In addition, of the ~5% processed, up to 60% of the broccoli head, which includes the base material and the stem is discarded during processing [1]. This is a significant underuse of edible biomass since the broccoli plant including the stem and the leaves is a rich source of protein, fiber, and phytochemicals such as polyphenols and glucosinolates (GLSs) [2,3]. Glucosinolates in particular are unique to cruciferous vegetables such as broccoli and are associated with several health benefits. Consumption of GLSs derived bioactive compounds such as isothiocyanates reduce the risk of cancer [4] and lower cholesterol [5]. The most abundant GLS in broccoli is glucoraphanin (4-methylsulfinylbutyl glucosinolate, GR) which is biologically inactive [6]. Bioactive compounds are formed upon hydrolysis of GR by the enzyme myrosinase (thioglucoside glucohydrolase, EC 3.2.3.147). Sulforaphane, an isothiocyanate that is formed as a result of GR hydrolysis in broccoli [7], has been found to be a potent bioactive against cancer [8], diabetes [9], and obesity [10], among other conditions.

Myrosinases are present in plants containing GLSs [7,11]. However, the conversion of GR to bioactive compounds occurs only when plant tissues undergo disruption during processing, cutting, and/or chewing, as myrosinase and GR are present in different cell compartments in the intact plant [11,12]. The rate of hydrolysis and the concentration of various products resulting from hydrolysis of GR depends on the reaction conditions (e.g., temperature, pH, ferrous ion concentrations) and presence of epithiospecifier protein (ESP) [13]. The production of sulforaphane (1-isothiocyanato-4-methylsulfinylbutane; SF) is favored at elevated temperature (above 50 °C) and natural pH, while sulforaphane nitrile synthesis is favored at lower temperature (below 50 °C), acidic pH, and the presence of ESP [7,14,15,16]. 

Due to the interest in sulforphane as a health promoting bioactive, many studies have examined cooking and processing methods to optimize sulforphane formation in broccoli and broccoli sprouts [7,14,17,18,19,20,21]. Pérez et al. [17] reported a significant increase in SF content by heating between 50 and 60 °C, with the optimal yield after immersion in water at 57 °C for 13 min and a decrease at higher temperatures (70 °C) due to thermal degradation of SF. Ghawi et al. [7] studied the effect of thermal processing (30–100 °C) on GR content, SF formation, and myrosinase activity in broccoli and reported significant increases in GR content after 4, 8, and 12 min heat treatment at 80 °C, and decrease in myrosinase activity at temperature >50 °C. Despite reduced myrosinase activity at temperatures >50 °C, the maximum SF formation was achieved after 8 and 12 min at 70 °C. The increase in sulforaphane yield under mild heating condition is mainly attributed to the selective inactivation of ESP; the protein co-factor that mediates conversion to sulforaphane nitrile instead of sulforaphane [16,22], although some studies suggest an increase in extractable GR after thermal processing at temperature as high as 100 °C. Nevertheless, the formation of SF is limited at temperatures >70 °C because of the sensitivity of both myrosinase and SF to thermal degradation [7,17]. 

While most studies examined the effects of heat on SF content, there are opportunities for enhancing SF content by use of other pre-processing treatments. The use of ultrasound in combination with moderate thermal treatment is considered attractive as ultrasound is known to increase the extractability of components from plant matrices [23] as well as enhance the activity of some enzymes and enzyme-substrate interaction. Under mild temperature conditions, ultrasound may increase enzyme activity by facilitating mass transfer and micro-mixing [24], which facilitate enzyme-substrate interaction and in some cases through changes in the conformation of enzymes, which potentially expose more active sites [25].

To our knowledge there is no published work on the combined effect of thermal and ultrasound treatment on the extractability of GR, SF formation and myrosinase activity in broccoli. It is expected that the interplay of all these factors influence SF yield. We hypothesized that the combination of ultrasound and moderate heat (thermosonication) would be more effective than heat treatment alone for disrupting plant tissue. This would enhance release of GR and increase the accessibility of myrosinase to GR, facilitating contact between myrosinase and GR due to increased diffusivity of these components. However, how thermosoncation would affect the activity of myrosinase is unknown. The aim of this study was to investigate the feasibility of thermosonication treatments for maximizing SF yield in broccoli puree. The effect of thermosonication on myrosinase activity and the extractability of GR were also investigated and compared with thermal processing without sonication.

## 2. Materials and Methods

### 2.1. Materials

Chemicals were purchased from Sigma-Aldrich (Sigma-Aldrich, St Louis, MO, USA) or Merck (Darmstadt, Germany) and were of analytical or HPLC grade, unless stated otherwise. Market fresh broccoli (cv. ‘Viper’) samples of ∼15 kg (3 batches each 5 kg on different days for three different treatment groups) were purchased from a local supermarket (Coles, Werribee, Victoria, Australia) and were stored at 4 °C for a maximum of 2 h prior to use. For each treatment temperature, the respective thermal and thermosonication experiments were conducted using the same batch of broccoli.

### 2.2. Preparation of Samples

Broccoli heads were washed with tap water and the florets were cut into 2–3 cm length. For each experimental run, 75 g of broccoli florets was weighed in a 500 mL glass container and 250 mL Milli-Q water was added. This allowed full coverage of the florets with water. This was necessary to facilitate the thermosonication treatments as it enabled the immersion of the tip of the ultrasound sonotrode into the sample. This ratio of sample/Milli-Q water was chosen based on preliminary experiments as higher sample/water ratios resulted in more solids, less homogeneity and consequently inhomogeneous acoustic energy densities delivered to the samples. In contrast, lower sample to water ratios led to over dilution of samples for adequate conversion into sulforaphane (data not shown). However, the results of the analyzed parameters following treatments were expressed on the basis of dry weight of the samples; calculated based on the moisture content of the samples (samples contained 3.04% dry matter) measured using a HR73 Halogen Moisture Analyzer (Mettler Toledo Ltd., Victoria, Australia).

### 2.3. Thermosonication and Thermal Treatments

**Figure 1:**
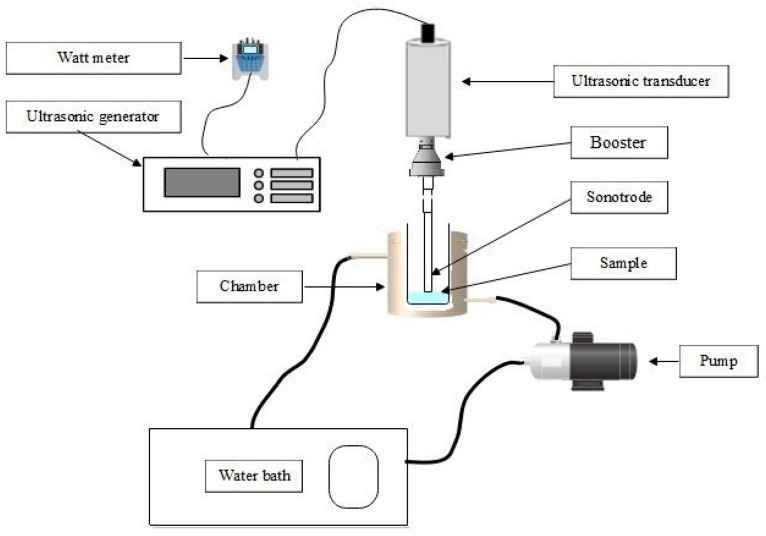
A schematic diagram of the thermosonication treatment set up.

The thermosonication experiments were conducted using an 18 kHz ultrasonic processor (UP500S, 500 W, Hielscher Ultrasonics, Teltow, Germany) with a titanium sonotrode (tip diameter 18 mm, length 70 mm). Due to an increase in the temperature during sonication, broccoli floret samples prepared as described in Section 2.2 were placed into a chamber with a water circulation system to maintain temperature at the target experimental temperature. Changes in temperature were monitored with a digital thermometer during the process. The maximum deviation from the target experimental temperature was 2 °C. The sonotrode was submerged ∼3 cm into the samples and the process was carried out at an ultrasonic power density of 0.60 ± 0.03 W/g for 3, 5, and 7 min at 40, 50, and 60 °C. A schematic diagram of the experimental set up is shown in Figure 1. Before treatment, samples were pre-heated to the experimental temperature in a thermostated water bath maintained at 50, 60, and 70 °C for about 10 min, for target temperatures of 40, 50, and 60 °C respectively. Samples were immersed in ice-water immediately after processing. For calculation of acoustic energy densities delivered to the samples, the following formula was used [26]:

(1) $$I=\frac{P}{W}$$

where *P* is ultrasound power (W), *W* is the weight of sample (g), and *I* is acoustic energy density (W/g). The actual power input during the sonication treatment was measured using a power meter (Watts Clever EW-AUS5001, Frankston, Australia) connected to the electric power outlet. Thermal treatment experiments were conducted for comparison at the same temperatures in a thermostated water bath. The temperature/times used were 40, 50 or 60 °C for 3, 5 or 7 min. Timing started after the florets reached the experimental temperature. Samples were cooled in ice-water immediately after processing. All experiments were conducted in triplicates.

### 2.4. Broccoli Puree Processing

Following thermosonication or thermal treatments, samples (75 g broccoli in 250 mL Milli-Q water) were pureed using a kitchen scale blender (Nutribullet pro 1200 series, LLC, Capital brands, Los Angeles, CA, USA). The purees were incubated for 2 h at 30 °C to allow myrosinase to hydrolyze glucoraphanin. Sub-samples were aliquoted for glucoraphanin and sulforaphane analysis and were frozen immediately and kept frozen at −18 °C until analyses. Sub-samples aliquoted for myrosinase activity assay were freeze-dried.

### 2.5. Glucoraphanin Extraction and Analysis

For the extraction of GR, two methods were initially examined: aqueous extraction or methanolic extraction. Previously, both water [27] and methanol [19] have been used for GR extraction. In the present study we wanted to compare the effects of using different extraction solvents to evaluate whether levels of extractable GR were influenced by the type of solvent. 

#### 2.5.1. Aqueous Extraction

The water extracts of broccoli samples were prepared according to the method previously described by Cai et al. [27] with some modifications. Four grams of frozen broccoli puree was mixed with 5 mL of boiling Milli-Q water and vortexed for 1 min. The mixture was then incubated in a boiling water bath for 5 min. After cooling in ice, samples were centrifuged at 5000× *g* for 15 min at 10 °C. Supernatants were collected and a second extraction of the residue was conducted with 5 mL of boiling Milli-Q water and the supernatant was collected. Finally, the supernatants were combined and evaporated to dryness with a vacuum spin dryer (SC250EXP, Thermo Fisher Scientific, CA, USA) at ambient temperature (~22 °C). Dried samples were dissolved appropriately in a solvent (acetonitrile/water (85:15, *v*/*v*) with 30 mM ammonium formate), filtered through a 0.22 µm membrane filter (Merck Millipore, Billerica, MA, USA) and analyzed by HPLC.

#### 2.5.2. Methanolic Extraction

The methanol extracts were prepared according to the method described by Pongmalai et al. [19] with some modifications. Ten grams of each sample was mixed with 17.96 mL of pre-heated methanol (70 °C) to obtain a 70:30 *v*/*v* methanol: water ratio. The mixture was then blended using an Ultra-Turrax T25 (JANKE and KUNKEL; IKA Labortechnik, Staufen, Germany) for one minute at 13,500 rpm. This was followed by incubation in a recirculating water bath (Thermoline, Wetherill Park, NSW, Australia) maintained at 70 °C for 30 min under continuous stirring at 100 rpm. Samples were then cooled in ice-water, centrifuged (5000× *g*/15 min/4 °C), and the supernatants were collected. The extraction was repeated twice. The supernatants were combined and the solvent was removed using vacuum spin dryer (SC250EXP, Thermo Fisher Scientific, Waltham, MA, USA) at ambient temperature (~22 °C). The residue was dissolved appropriately in solvent (acetonitrile/water (85:15, *v*/*v*) with 30 mM ammonium formate), filtered through a 0.22 µm membrane filter (Merck Millipore, Billerica, MA, USA) and analyzed by a HPLC method.

#### 2.5.3. Analysis and Quantification of Glucoraphanin

Quantification of glucoraphanin was carried out by using an Alliance HPLC instrument (Waters Corporation, Milford, MA, USA) equipped with Photo Diode Array Detector 2998 in accordance with the method of Cai et al. [27] with some modifications. A HPLC column–Luna^®^ 3 µM Hydrophilic Interaction Liquid Chromatography (HILIC) 200° A (100 × 4.6 mm; Phenomenex, Torrance, CA, USA) was used for the analysis at a column temperature of 25 °C. The mobile phase consisted of an acetonitrile/water (85:15, *v*/*v*) with 30 mM ammonium formate (solution A) and acetonitrile (solution B) with the following isocratic flow program: solution A 70%; solution B 30%. Other chromatographic conditions included a constant flow rate of 2.0 mL/ min, an injection volume of 100 µL, a run time of 8 min, and detection wavelength of 235 nm. The identification of the glucoraphanin peak was based on the retention time and the chromatographic spectra of an authentic glucoraphanin standard. The concentrations of glucoraphanin were calculated using a standard curve developed with a glucoraphanin standard (Cayman Chemical Company, Ann Arbor, MI, USA) and was expressed as micrograms per gram dry weight (µg/g DW) of broccoli. The results were expressed as normalized glucoraphanin content by dividing the glucoraphanin amount after thermal or thermosonication treatment by glucoraphanin amount in untreated samples. The results were normalized as there was batch to batch variation in broccoli composition.

### 2.6. Sulforaphane Extraction and Analysis

#### 2.6.1. Extraction

The extraction and analysis of sulforaphane was conducted as described in Cai et al. [28] with some modification. Briefly, 4 g of frozen sample was mixed with 2 mL of Milli-Q water and vortexed for 1 min. Then, 20 mL of ethyl acetate was added and the mixture was sonicated using a sonication bath (IDK technology Pty Ltd., VIC, Australia) for 5 min followed by shaking for 20 min at 4 °C. The mixture was centrifuged at 5000× *g* for 15 min at 10 °C and the supernatant was collected. A second extraction was carried out by adding 15 mL ethyl acetate to the residue. The supernatants from the two extractions were combined and dried using a vacuum spin dryer (SC250EXP, Thermo Fisher Scientific, Waltham, MA, USA) at room temperature (22 °C). All samples were dissolved appropriately in 30% acetonitrile, filtered through a 0.22 µm membrane filter (Merk Millipore, Billerica, MA, USA) and analyzed by UPLC.

#### 2.6.2. Analysis and Quantification

The sulforaphane content of the samples was analyzed using an Acquity™ Ultra Performance liquid chromatography (UPLC) system (Waters Corporation, Milford, MA, USA), which is equipped with a binary solvent delivery manager and a sample manager. Chromatographic separations were performed on a 2.1 mm × 50 mm, Acquity BEH C18 chromatography column. The mobile phase A and B were 0.1% Formic acid in Milli-Q water and 0.1% Formic acid in acetonitrile, respectively. The gradient elution system consisted of mobile phase A (0.1% Formic acid in Milli-Q water) and B (0.1% Formic acid in acetonitrile) and separation was achieved using the following gradient: 0–2 min, 10% B; 2–5 min, 20% B; 5–10 min, 10% B. The column temperature was kept constant at 30 °C. The flow rate was 0.350 mL/min and the injection volume was 10 µL. The concentration of sulforaphane was calculated based on a standard curve developed with an authentic sulforaphane standard (DL–sulforaphane, Sigma–Aldrich, St. Louis MO, USA), and the results were expressed as micrograms per gram dry weight (DW) (µg/g DW) of broccoli. The results were reported as normalized sulforaphane content by dividing measured sulforaphane concentration after thermal or thermosonication treatment by measured sulforaphane concentration in untreated samples to allow for comparisons between different batches of broccoli samples.

### 2.7. Determination of Myrosinase Activity

#### 2.7.1. Myrosinase Extraction

Myrosinase extraction was conducted following the method described by Ghawi et al. [7] with modification. Briefly, 500 mg freeze-dried broccoli powder was suspended in 15 mL sodium phosphate buffer (100 mM, pH 6.5) and blended using an Ultra-Turrax T25 (JANKE and KUNKEL; IKA Labortechnik, Staufen, Germany) at 13,500 rpm for 1 min. The mixture was stirred for 1 h at 4 °C and then centrifuged at 10,000× *g* for 15 min at 4 °C. The supernatant was recovered and was subjected to ammonium sulfate precipitation at 80% saturation. The precipitate was dissolved in 1 mL sodium phosphate buffer (100 mM pH 6.5) and used as enzyme extract for myrosinase activity assay.

#### 2.7.2. Myrosinase Activity Assay

Myrosinase activity was determined as the amount of glucose released from the hydrolysis of sinigrin by myrosinase after 15 min incubation at 30 °C [7,19]. One mL reaction mixture containing 250 µL enzyme extract, 750 µL sodium phosphate buffer (100 mM, pH 6.5), 1 mM EDTA, 3 mM MgCl_2_, and 1 mM sinigrin as substrate was prepared. All chemicals were purchased from Sigma (St. Louis, MO, USA). After incubation at 30 °C for 15 min the reaction was stopped by placing samples in boiling water (100 °C) for 5 min. The mixture was centrifuged at 25,000× *g* for 10 min at 4 °C and the amount of glucose released was quantified using Glucose (HK) assay kit (Sigma, St. Louis, MO, USA) according to the procedure provided in the kit. One unit of myrosinase activity was defined as microgram glucose per min released during the myrosinase catalyzed hydrolysis of sinigrin under the assay condition. The result was presented as relative activity of myrosinase i.e., (A/Ao) with A denoting myrosinase activity after thermal or thermosonication treatment and Ao denoting myrosinase activity in the untreated sample.

### 2.8. Statistical Analysis

Statistical analysis of the data was performed using SPSS (version 18.0 for Windows, SPSS, Inc., Chicago, IL, USA) software and mean value ± standard deviation of three replications were reported. The data normality and homogeneity of variances were tested using Shapiro-Wilk’s and the Levene’s tests, respectively. Significant differences in mean values were calculated using Duncan’s post hoc test. Differences between mean values were considered significant at *p* < 0.05.

## 3. Results and Discussion

### 3.1. The Stability of Broccoli Myrosinase Subject to Thermosonication and Thermal Processing

**Figure 2:**
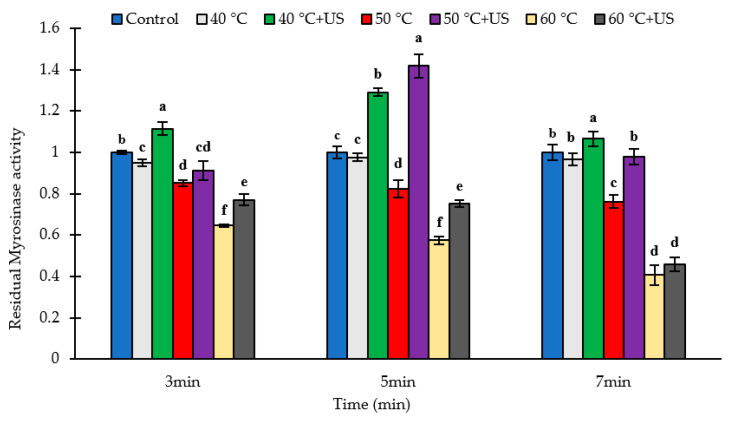
The residual activity of myrosinase in broccoli puree after thermal/thermosonication pre-treatments of broccoli floret. Letters above each bar indicate the results of Duncan’s test; Different letters within the same group indicate significant difference (*p* < 0.05).

The effects of thermal and thermosonication treatments on the activity of broccoli myrosinase are presented in Figure 2. The initial amount of myrosinase activity was on average 88.7 U/g DW for batch 1, 89.3 U/g DW for batch 2, and 110.9 U/g DW for batch 3 broccoli samples (Table S1 in the Supplementary Materials). There was a significant batch to batch variation in the myrosinase activity of the broccoli samples. The thermal inactivation of broccoli myrosinase commenced at 40 °C with 4.9, 2.4 and 3.2% inactivation after 3, 5 or 7 min heating, respectively, compared to untreated broccoli (*p* < 0.05). This is in agreement with the results of Ghawi et al. [7] who reported that the inactivation of myrosinase in broccoli was initiated at 40 °C, and its activity decreased by more than 80% after 12 min at 60 °C with the activity completely lost after heating at 80 °C for 12 min. As would be expected, the degree of myrosinase inactivation increased with increase in temperature and treatment time. Thermal treatment at 50 °C and 60 °C decreased myrosinase activity by 14.9–23.8% and 35.4–59.3% respectively compared to untreated broccoli, depending on duration of thermal treatment (Figure 2). Under the conditions examined, the maximum inactivation was observed after 7 min heating at 60 °C (59.3% decrease). 

In contrast, thermosonication treatment at 40 °C for various times led to significant increases (*p* < 0.05) in myrosinase activity of 6.6–29.1% compared to untreated broccoli. The impact of thermosonication treatment at 50 °C was dependent on the treatment time; 5 min treatment resulted in a ~40% higher (*p <* 0.05) myrosinase activity compared to control whereas 3 min treatment resulted in a slight decrease in activity (*p* <0.05) with no difference in activity observed between the control and samples treated for 7 min (Figure 2). On the other hand, thermosonication treatments at 60 °C resulted in a slight but significant (*p* < 0.05) decrease in the activity of myrosinase compared to the untreated control for all treatment times. However, the level of activity decrease after thermosoncation at 60 °C was significantly lower (*p* < 0.05) than those observed for the corresponding thermal treatments at the same time/temperature condition except for the 7 min treatment. The residual myrosinase activity in samples thermosonicated at 60 °C for 7 min was 5.4% higher than the corresponding values for thermally treated samples at the same condition, although the difference was not statistically significant (*p* > 0.05).

There are conflicting reports on the thermal stability of broccoli myrosinase, partly due to the difference in the media in which the studies are conducted, varietal differences and the method of heat treatment. Ludikhuyze et al. [29] reported that broccoli myrosinase inactivation in a buffer system (0.1 M phosphate buffer at pH 6.55, which corresponds to the pH of the fresh broccoli juice) started at 30 °C and more than 90% of the activity was lost after 3 min treatment at 60 °C. Van Eylen et al. [22] reported that broccoli myrosinase was stable until 45 °C, when vacuum packed broccoli heads were heated in a water bath, and its activity was reduced by more than 95% after a 10 min treatment at 70 °C. Ghawi et al. [7] studied the effect of thermal treatments (30–100 °C) using the sous vide cooking method on myrosinase activity in broccoli heads and observed more than 80% loss after 4 min at 90 °C, and only 6% residual activity after 8 min at 80 °C. Contrary to previous studies, Pérez et al. [17] reported a significant increase in myrosinse activity in broccoli floret during blanching by putting broccoli florets directly into a thermostatic bath containing deionized water at temperatures ranging from 46–74 °C for 3–17 min with the maximum activity observed after heating at 70 °C for 17 min. They attributed that to the increased extractability of myrosinase with increase in blanching temperatures. 

An interesting result in our study was that there was in general higher residual myrosinase activity in broccoli samples subjected to thermosonication compared to the corresponding heated samples, with few exceptions where the difference was not statistically significant. While thermosonication treatment using low frequency high intensity ultrasound might be expected to cause a higher level of inactivation of enzymes compared to thermal treatment alone [25], other factors including the intensity of the ultrasonic treatment and the effects of ultrasound on the plant matrix can impact the observed effect on enzyme activity. Ultrasound can have dual effects on enzyme activity in tissue systems. On one hand, the strong shear force generated during acoustic cavitation produces mechanical disruption of the broccoli matrix and cell damage with the consequent release of myrosinase from myrosin cells [18] resulting in increased activity. On the other hand, the extreme localized increase in temperature (500 K) and pressure (50 MPa), the strong shear and microstreaming and the generation of free radicals that accompany cavitation can cause changes in the structure and conformation of enzymes leading to inactivation [25]. Synergistic inactivation of enzymes by ultrasound combined with mild heat has been reported in several studies [23,25,30]. The measured enzyme activity depends on whether extraction or inactivation is the dominant effect. Thus, the observed higher residual myrosinase activity in the sonicated samples does not necessarily imply that thermosonication has less inactivating effect on myrosinase compared to thermal treatment under the corresponding heat treatment conditions, rather sonication induced enhanced extraction is possibly the dominant effect.

### 3.2. Effect of Thermosonication and Heat on Sulforaphane Yield

**Figure 3:**
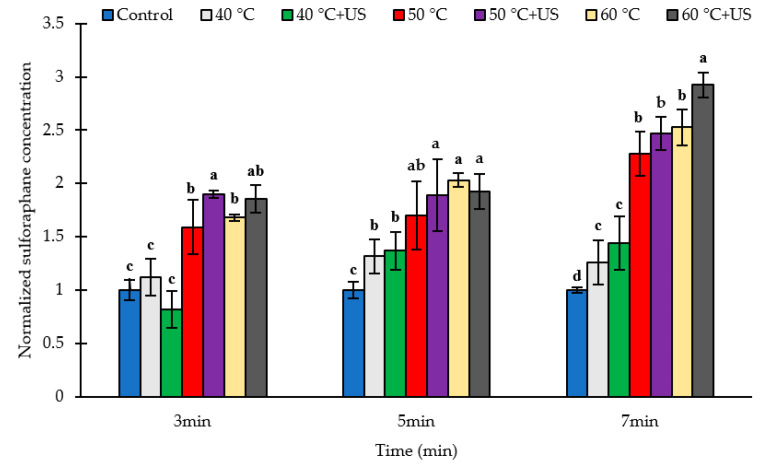
The normalized sulforaphane yield (Sulforaphane content in treated sample/sulforaphane content in untreated control) in broccoli puree after thermal/thermosonication pre-treatments of broccoli floret. Letters above each bar indicate the results of Duncan’s test; Different letters within the same group indicate significant difference (*p* < 0.05).

The effects of thermal treatment (40, 50, or 60 °C for 3, 5, or 7 min) and thermosonication (18 kHz) on the yield of the bioactive sulforaphane (SF) in broccoli puree after incubation for 2 h at 30 °C are presented in Figure 3 and Table S1 of the Supplementary Materials. The SF content in non-treated broccoli puree were on average 195.9 µg/g DW, 180.6 µg/g DW and 592.1 µg/g DW for batch 1, batch 2 and batch 3 samples respectively (Table S1 in Supplementary Materials). The higher level of sulforaphane in the untreated batch 3 samples could be due to the higher myrosinase activity in this sample (Section 3.1.). Heating alone for 3, 5, and 7 min at 40 °C increased SF by 12.9, 31.7, and 26.0% respectively, compared to raw broccoli, although the increase for 3 min treatment was not statistically significant (*p* > 0.05). Thermal treatment at 50 °C or 60 °C for 3, 5 or 7 min significantly increased (*p* < 0.05) SF yield when compared to untreated broccoli. The SF contents were 68.1–152.6% higher after treatment at 60 °C compared to the untreated control whereas the SF contents were 59 to 128% higher than control in the case of the 50 °C heat treatments. Nevertheless, the difference in the relative increase of sulforaphane content between 50 and 60 °C was not statistically significant (Figure 3). The maximum SF concentration among the studied conditions was achieved after 7 min heat treatment at 60 °C (Table S1). This may not represent the absolute maximum since increase in treatment time or temperature may result in a higher sulforaphane yield, although earlier studies [7] indicate that longer treatments at 60 °C may result in substantial inactivation of myrosinase and hence lower sulforaphane yield. 

Except in some cases such as 40 °C for 3 min (Figure 3), thermosonication increased SF yield by 36.7–192.6% compared to untreated samples and 5.06–40.0% in comparison to corresponding heat-treated samples without ultrasound, although the difference between the two was statistically significant only for the 3 min treatment at 50 °C and the 7 min treatment at 60 °C. The maximum SF content among the studied conditions was achieved using thermosonication treatment at 60 °C for 7 min, which represented a 192.6% increase compared to untreated broccoli florets. That was 15.8% higher than the corresponding thermal treatment indicating the potential of thermosonication for enhancing sulforaphane yield over and above the effect of heat. Further increase in treatment temperature or time may result in higher sulforaphane yield, although inactivation of myrosinase under more intense thermosonication condition may have the opposite effect. Notwithstanding, further optimization of the thermosonication treatment condition is required in order to realize the full potential of the technology and confirm indeed whether the technology enables better sulforaphane yield compared to thermal treatment alone.

From the results it is evident that thermal treatment at 50 to 60 °C significantly increases the SF content in broccoli. In agreement with these observations, Ghawi et al. [7] reported SF formation in broccoli floret increased by 6 times compared to the raw samples, after heating for 12 min at 70 °C, where they used the sous vide cooking method for thermal treatment of broccoli florets. Similar findings were reported by Sarvan et al. [31], Bello et al. [14], Pongmalai et al. [19], Pongmalai et al. [18], and Pérez et al. [17]. Increase in SF content in broccoli subjected to mild heat treatment is commonly attributed to thermal inactivation of the thermolabile epithiospecifier protein (ESP), which acts as non-catalytic cofactor for myrosinase that promotes the formation of SF-nitrile from GR instead of SF [11]. Matusheski et al. [15] observed a significant inactivation of ESP in broccoli after 5 and 10 min heating at 40 °C and 50 °C, respectively, and complete inactivation after 5 min heating at 60 °C, which was accompanied by higher SF formation. Nevertheless, the heat-induced increase in extractability and accessibility of GR [7] may also contribute to the observed higher yield of SF in mild-heat-treated broccoli. The results of this study also showed the potential of thermosonication to enhance SF yield in broccoli puree over and above the effects of mild heat. This could be due to cavitation induced cell disruption that facilitate the release and accessibility of glucoraphanin and subsequent myrosinase-glucoraphanin interaction. As discussed in Section 3.3 below, ultrasound results in higher amount of extractable GR, indicating that the treatment results in increased GR release, and improved accessibility for interaction with myrosinse, resulting in higher SF production.

It is important to note that sulforaphane yield did not correlate with the degree of inactivation of myrosinase and the remaining residual activity. On the contrary, higher sulforaphane yield was observed under conditions of significant myrosinase inactivation. This could be attributed to the inactivation of ESP, thus providing a suitable condition for the synthesis SF from GR as opposed to SF-nitrile and/or increased in situ residual myrosinase activity at elevated temperatures [20] and increased release and accessibility of GR. In agreement with our results, Sarvan et al. [31] observed a significantly higher SF content in broccoli florets after 2 min steaming (about 85 °C) compared to raw broccoli samples, although the residual myrosinase activity was only 2.5%.

### 3.3. Effect of Heat and Thermosonication Treatments on Glucoraphanin Content

**Figure 4:**
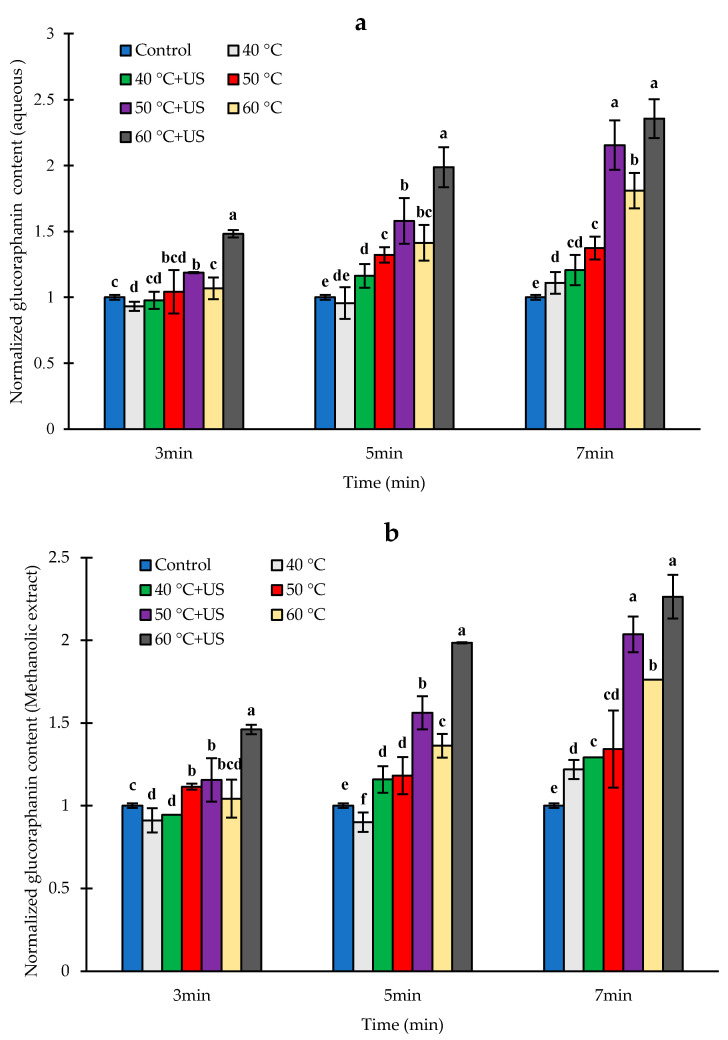
The normalized residual level of glucoraphanin contents (glucoraphanin content in treated samples/glucoraphanin content in untreated control) in aqueous (**a**) and methanolic (**b**) extracts from broccoli puree after thermal/thermosonication pre-treatments of broccoli florets and conversion to sulforaphane. Letters above each bar indicate the results of Duncan’s test; Different letters within the same group indicate significant difference (*p* < 0.05).

The GR content of aqueous and methanolic extracts of untreated broccoli florets after pureeing and incubation were on average 355.5 ± 15.2 and 405.2 ± 41.9 µg/g DW respectively (Table S1 in Supplementary Materials), which was a significant decrease compared to the typical amount of ~1500 µg/g DW or ~3.4 µmol/g DW for intact broccoli florets from the same source observed in our previous studies [28,32]. The glucoraphanin content of broccoli is dependent on factors such as cultivar and growing condition and the reported values range from 0.3 to 38.4 µmol/Kg DW [33]. For instance, Pérez, et al. [17] reported ~ 12.6 µmol/g DW, Jones et al. [34] reported 28.6 µmol/g DW for Marathon cultivar and 13.1 µmol/g DW for Booster™ cultivar and Sarvan et al. [35] reported 181 µmol/100 g fresh weight (equivalent to 18.1 µmol/g DW). The significant decrease in GR content of broccoli after pureeing and incubation could be attributed to enzymatic conversion of GR into sulforaphane and other metabolites. Glucosinolates including GR may undergo non-enzymatic degradation and conversion at acidic, alkaline, or extreme heat (>100 °C) conditions or in the presence of certain chemicals [36]. However, at the natural pH of broccoli and under mild processing conditions as in this study, the main mechanism of degradation is enzymatic hydrolysis mainly to sulforaphane or sulforaphane nitrile in the presence of active ESP [33]. The difference between the GR content of aqueous and methanolic extracts was not statistically significant (*p* > 0.05). The relative changes in the concentrations of aqueous and methanolic extracts of glucoraphanin (GR) after thermal and thermosonication treatments followed by pureeing and incubation are shown in Figure 4a,b, respectively. A significant amount of unconverted glucoraphanin remained in all the samples, possibly due to incomplete conversion during the short incubation time in addition to increased release of glucoraphanin from broccoli tissue counteracting the effect of GR conversion.

The measured residual GR content of broccoli samples after heat treatment for 3 or 5 min at 40 °C were slightly lower than untreated samples; although these differences were statistically significant (*p* < 0.05) only for the 3 min treatment. Similarly, treatments at 50 or 60 °C for 3 min did not have significant effects on GR content of broccoli samples (*p* > 0.05), while other thermal treatment conditions increased the measured residual level of GR significantly (*p* < 0.05) compared to untreated samples. This is despite the higher sulforaphane yield compared to control under these conditions (Figure 3), indicating that heat treatment enhances the release, extractability and subsequent accessibility of glucoraphanin for enzymatic conversion. 

With regard to thermosonication, a significant increase in the residual content of GR compared to the untreated samples was observed except for the treatments at 40 °C for 3 min (Figure 4a,b). The residual level of GR, after 5 or 7 min thermosonication at 50 °C were 56.2% and 103.2% higher (*p* < 0.05) respectively than those in control. The highest residual level of GR was observed after thermosonciation at 60 °C for 7 min (126.3% increase) followed by those that were thermosonicated at 50 °C for 7 min (103.6% increase compared to untreated samples), with no statistically significant difference between the two. Interestingly, the highest level of conversion to sulforaphane also occurred in samples that were thermosonicated for 7 min at 60 °C. Despite this high level of conversion to sulforaphane, there was substantial amount of glucoraphanin (837.7 µg/g DW or 1.9 µmol/g DW) remaining in the samples, which is more than 56% of the typical measurable glucoraphanin content in intact broccoli florets (~1500 µg/g DW or 3.4 µmol/g DW) of the same type as mentioned above. The sulforaphane yield in samples thermosonicated for 7 min at 60 °C was 1734.0 µg/g DW or 9.8 µmol/g DW. Assuming 1 to 1 stochiometric conversion of glucoraphanin to sulforaphane, the maximum level of sulforaphane that can be produced based on the typical measurable level of glucoraphanin in intact broccoli floret is ~3.4 µmol/g DW. In this case, 9.8 µmol/g DW was observed, which is about 2.9 times more while about 1.9 µmol/g DW glucoraphanin remained unconverted in the sample. This indicates that the total amount of glucoraphanin in the broccoli sample is at least 11.7 µmol/g DW i.e., 9.8 µmol/g DW which was converted to sulforaphane plus 1.9 µmol/g DW glucoraphanin that remained in the sample. It can be concluded that (1) the measured level of glucoraphanin in untreated broccoli using current methods gives only an indication of the amount of easily accessible and extractable glucoraphanin in broccoli, and (2) processing of broccoli by heat and ultrasound enables the release of possibly bound glucoraphanin and enhance conversion to sulforaphane. A high degree of correlation (r = 0.77–0.84 based on aqueous extraction) was observed between residual GR and SF contents in the processed samples, indicating the importance of accessible GR on SF yield. Further studies on the impact of thermal and ultrasonic processing on the extraction yield of glucoraphanin under conditions that inhibit myrosinase catalyzed conversion would enable a better understanding of the impact of these processes on the extractability of glucoraphanin and its consequence on myrosinase-glucoraphanin interaction and sulforaphane yield.

The observed increase in GR extractability with increasing temperature of heat treatment of broccoli florets can be attributed mainly to heat-induced structural modification of the broccoli matrix, which facilitates the release of GR [7,17,19,37]. Živković et al. [36] suggested that at elevated temperatures, there is degradation of plant matrix and cellular structures, which increases cell permeability. 

In agreement with our observation, Sarvan et al. [31] reported 80% increase in GR concentration in broccoli after 2 min steaming. Ghawi et al. [7] observed 15, 11, and 5% increase in GR content in broccoli after 4, 8, and 12 min heat treatment at 80 °C. Pérez et al. [17] investigated the optimization of blanching step to maximize sulforaphane synthesis in broccoli at temperatures ranging from 46–76 °C for durations of 3–17 min. They found a trend of increasing GR content as temperature increased, with the maximum content observed after 15 min heating at 70 °C which was 93% higher compared to fresh broccoli.

Our results showed that a combination of ultrasound with heat treatment (at 50 and 60 °C for 5 and 7 min) improved the release of GR from broccoli matrix over and above the effect of heat treatment alone. This could be due to mechanical disruption of the plant cell wall and plant matrix, which enhances the release of intracellular content (plant constituents) by extraction [23]. The micro-streaming, micro-jets, and hydrodynamic shock waves generated during acoustic cavitation that creates an extreme shear forces, are believed to be responsible for mechanical rupture of plant tissue during ultrasonication [38]. The mass transfer increases due to acoustic cavitation can also facilitate diffusion rates, subsequently increasing GR extractability. Aguilar-Camacho, Welti-Chanes, and Jacobo-Velázquez [39] observed 795% increase in GR extractability in broccoli florets by ultrasound treatment (400 W, 24 kHz, 100 μm amplitude, 20 min). Similarity, Pongmalai et al. [19] found 87% increase in GR content of steamed white cabbage by using combined ultrasound-assisted extraction (37 kHz, 0.03 W/g, 40 min) and microwave-assisted extraction after 2 min steaming, compared with those in fresh cabbages without steaming indicating the potential of ultrasound for enhancing the extraction of GR as observed in this study.

## 4. Conclusions

This study showed that thermosonication treatment of broccoli florets at 50 to 60 °C enhances the yield of sulforaphane in broccoli puree compared to control. The highest sulforaphane yield was observed after 7 min thermosonication treatment of broccoli floret at 60 °C, which resulted in 2.9 times more yield of sulforaphane compared to untreated broccoli. Thermal processing at the same condition (60 °C/7 min) resulted in 2.5 more sulforaphane yield compared to the untreated control. The underlying mechanism appears to be not only the inactivation of ESP but also heat and ultrasound induced release and enhanced accessibility of glucoraphanin for myrosinase catalyzed conversion into sulforaphane. The results indicate that thermosonication treatment may improve sulforaphane yield over and above the effect of heat under the same condition, although further experiments at various ultrasonication conditions are required to confirm that. The study showed that thermosonication at relatively low power input (0.6 W/g) enhances sulforaphane yield in broccoli indicating that the process can be used for cost effective conversion of broccoli biomass including low value second grade produce into high value ingredients enriched with sulforaphane for applications in food, nutraceutical, and pharmaceutical products. Further improvement in sulforaphane yield is possible via optimization of the ultrasonic processing condition.

## Data Availability

Data is available on request.

## Supplementary Materials

The raw sulforaphane, glucoraphanin and myrosinase activity data are available online at https://www.mdpi.com/2218-273X/11/2/321/s1, Table S1. Effects of thermal and thermosonication processing on myrosinase activity, glucoraphanin content, and sul-foraphane yield in broccoli puree.
