# Supplementary material for: Thermosonication for the Production of Sulforaphane Rich Broccoli Ingredients
Source: Biomolecules. 2021 Feb 20;11(2):321. doi: 10.3390/biom11020321 (PMC7923798; doi:10.3390/biom11020321)
Supplement: Supplementary file 1 [file biomolecules-11-00321-s001.pdf]

Table S1. Effects of thermal and thermosonication processing on myrosinase activity, glucoraphanin content, and sulforaphane yield in broccoli puree.

			Glucoraphanin (aqueous extract; µg/g DW)	Glucoraphanin (methanolic extract; µg /g DW)	Sulforaphane (µg/g DW)	Myrosinase activity (U/gDW)
Thermal Treatment	40 °C	Untreated broccoli	355.5±15.2^{jlr}	405.3±41.9^{nop}	196.0±57.9^{mn}	88.2±0.7^{gh}
		3 min	331.3±12.0^{lms}	369.3±29.7^{op}	219.7±34.5^{mno}	83.8±1.4^{j}
		5 min	340.1±42.5^{ikmqr}	364.6±23.7^{p}	258.1±30.8^{lm}	86.0±1.7^{hij}
		7 min	394.6±29.1^{gjq}	493.9±23.5^{ikl}	246.9±40.0^{lmn}	85.3±2.6^{hij}
	50 °C	Untreated broccoli	355.5±15.2^{jrs}	405.3±41.9^{nop}	180.6±31.6^{no}	89.3±2.5^{dfghj}
		3 min	370.4±58.5^{gklmp}	451.4±7.4^{mn}	287.5±45.5^{klm}	76.0±1.4^{k}
		5 min	470.0±20.6^{fn}	478.7±45.3^{iklmnr}	307.3±57.4^{hiklm}	73.4±3.7^{klm}
		7 min	488.6±30.8^{ef}	543.8±94.4^{efghkm}	412.3±37.6^{fg}	68.0±2.8^{mn}
	60 °C	Untreated broccoli	355.5±15.2^{jks}	405.3±41.9^{nop}	592.6±99.2^{e}	110.9±4.3^{b}
		3 min	379.9±29.5^{hijp}	422.2±46.5^{lmnop}	996.4±18.1^{d}	71.6±0.6^{lm}
		5 min	502.5±48.3^{eno}	551.9±28.8^{ghr}	1202.8±38.7^{c}	63.7±2.2^{n}
		7 min	643.4±47.5^{cd}	714.0±0.0^{d}	1496.96±100.81^{b}	45.1±5.4^{o}
Thermosonication (18 kHz, 500 W) Treatment	40 °C	Untreated broccoli	355.5±15.2^{js}	405.3±41.9^{nop}	196.0±57.9^{mno}	88.2±0.7^{fgh}
		3 min	347.1±23.2^{jm}	382.4±10.9^{op}	160.2±34.1^{o}	98.2±2.8^{c}
		5 min	413.5±32.0^{gi}	469.3±32.8^{klmn}	268.0±33.9^{klm}	113.8±1.7^{b}
		7 min	429.0±40.9^{fgho}	523.4±15.1^{hi}	282.3±49.0^{klm}	93.9±3.2^{cd}
	50 °C	Untreated broccoli	355.5±15.2^{js}	405.3±41.9^{nop}	180.6±31.6^{no}	89.3±2.5^{dfgh}
		3 min	422.1±1.3^{g}	468.2±53.5^{ilkmnr}	343.2±6.0^{i}	81.6±4.2^{ijk}
		5 min	561.4±61.6^{de}	633.0±40.6^{efq}	341.4±61.2^{ghik}	126.7±5.2^{a}
		7 min	766.2±66.5^{ab}	825.2±43.9^{bc}	446.5±28.1^{f}	87.4±3.4^{efghij}
	60 °C	Untreated broccoli	355.5±15.2^{js}	405.3±41.9^{nop}	592.6±99.2^{e}	110.9±4.3^{b}
		3 min	526.9±10.1^{e}	591.9±11.5^{fgq}	1101.0±75.5^{c}	85.5±3.0^{ghij}
		5 min	706.7±54.1^{bc}	804.4±1.2^{c}	1140.3±98.5^{c}	83.3±2.0^{ij}
		7 min	837.7±52.4^{a}	917.3±53.6^{ab}	1734.1±70.0^{a}	51.0±3.7^{o}
